# Effects of early dialysis on the outcomes of critically ill patients with acute kidney injury: a systematic review and meta-analysis of randomized controlled trials

**DOI:** 10.1038/s41598-019-54777-9

**Published:** 2019-12-04

**Authors:** Wei-Ting Lin, Chih-Cheng Lai, Shen-Peng Chang, Jian-Jhong Wang

**Affiliations:** 1 Department of Orthopedic, Chi Mei Medical Center, Tainan, Taiwan; 2 Department of Physical Therapy, Shu Zen Junior College of Medicine and Management, Kaohsiung, Taiwan; 3 Department of Internal Medicine, Kaohsiung Veterans General Hospital, Tainan Branch, Tainan, Taiwan; 4 Yijia Pharmacy, Tainan, Taiwan; 5 Department of Internal Medicine, Chi Mei Medical Center, Chiali, Tainan, Taiwan

**Keywords:** Bacterial infection, Acute kidney injury

## Abstract

The appropriate timing for initiating renal replacement therapy (RRT) in critically ill patients with acute kidney injury (AKI) remains unknown. This meta-analysis aims to assess the efficacy of early initiation of RRT in critically ill patients with AKI. The Pubmed, Embase and Cochrane databases were searched up to August 13, 2019. Only randomized controlled trials (RCTs) comparing the effects of early and late RRT on AKI patients were included. The primary outcome was 28-day mortality. Eleven RCTs including 1131 and 1111 AKI patients assigned to early and late RRT strategies, respectively, were enrolled in this meta-analysis. The pooled 28-day mortality was 38.1% (431/1131) and 40.7% (453/1111) in the patients assigned to early and late RRT, respectively, with no significant difference between groups (risk ratio (RR), 0.95; 95% CI, 0.78–1.15, *I*^2^ = 63%). No significant difference was found between groups in terms of RRT dependence in survivors on day 28 (RR, 0.90; 95% CI, 0.67–1.25, *I*^2^ = 0%), and recovery of renal function (RR, 1.03; 95% CI, 0.89–1.19, *I*^2^ = 56%). The early RRT group had higher risks of catheter-related infection (RR, 1.7, 95% CI, 1.01–2.97, *I*^2^ = 0%) and hypophosphatemia (RR, 2.5, 95% CI, 1.25–4.99, *I*^2^ = 77%) than the late RRT group. In conclusion, an early RRT strategy does not improve survival, RRT dependence, or renal function recovery in critically ill patients with AKI in comparison with a late RRT strategy. However, clinicians should be vigilant because early RRT can carry higher risks of catheter-related infection and hypophosphatemia during dialysis than late RRT.

## Introduction

Acute kidney injury (AKI) is a common complication in critically ill patients, and the incidence of AKI patients requiring dialysis has also increased recently^1–3^. The development of AKI can be associated with increased morbidity, mortality and health-care costs^1,4,5^. Renal replacement therapy (RRT) remains the primary supportive strategy in the management of critically ill patients with severe AKI. However, in spite of improvements in RRT technology, such as the advent of continuous renal replacement therapy (CRRT) for patients with unstable hemodynamics, the mortality of patients with AKI requiring RRT remains high^6–8^. Several issues need to be addressed to improve the outcomes of these critically ill patients, such as the appropriate time to initiate RRT, the optimal intensity, and the choice of modality for RRT.

In common practice, RRT is usually initiated for acute management of life-threatening complications of AKI such as severe hyperkalemia, pulmonary edema, refractory metabolic acidosis, uremic pericarditis, and uremic encephalopathy^9^. Beyond these indications however, the appropriate timing for initiating RRT in critically ill patients is unknown. Early initiation of RRT is supposed to achieve better fluid and electrolyte balance, superior acid-base homeostasis, and more efficient removal of uremia toxins than standard therapy. Through these mechanisms, early RRT may help prevent AKI -associated kidney-specific or other vital organ injuries^10–13^. However, early RRT carries risks of several adverse events including vascular access placement-associated complications, catheter-related infections, bleeding due to the use of anticoagulants, too rapid changes in electrolytes, unnecessary clearance of important medications, delayed recovery of renal function and increased costs^13,14^. Several randomized controlled trials^15–23^ (RCTs) were conducted to find the optimal timing of RRT for critically ill AKI patients, but no consistent results were found. In 2018, one large RCT^24^ focused on patients with septic shock and severe AKI in the IDEAL-ICU trial found no significant difference in 90-day mortality between patients with early and delayed initiation of RRT. Their findings were consistent with those in another multicenter RCT by the AKIKI study group^22^, but were contrary to the findings of a recent single-center RCT in the ELAIN trial^23^. All of these findings indicate uncertainty about the usefulness of early RRT in critically ill patients. Although this issue had been discussed in one meta-analyses^25^ recently, we aimed to conduct an updated systematic review and meta-analysis of RCTs to assess the efficacy of early initiation of RRT in critically ill AKI patients.

## Materials and Methods

### Study search and selection

This systematic review and meta-analysis were conducted according to the preferred reporting items for systematic reviews and meta-analyses (PRISMA) statement (Supplemental Table 1). All clinical studies were identified by a systematic review of the literature in the PubMed, Embase, and Cochrane databases until August 13, 2019 using the following Mesh terms – “earl*”, “accelerat*”, “acute kidney”, “acute renal”, “anuria”, “oliguria”, “acute renal failure”, “anuria”, “oliguria”, “organ failure”, “dialy*”, “renal replacement”, “hemodialysis”, “hemofiltration”, “hemodiafiltration”, “RCT*” and “random*”. We excluded observation studies, case reports or case series, studies enrolling pediatric patients, and conference abstracts, and therefore, only RCTs that compared the clinical efficacy of early RRT and late RRT for critically ill adult patients with AKI were included. In addition, we searched all references in the relevant articles and reviews for additional eligible studies. Two reviewers (Chang & Wang) searched and examined publications independently to avoid bias. When they disagreed, another author (Lai) resolved the issue. The data included authors, year of publication, study design and duration, study population, sites of study, disease severity, indications for early RRT, and outcomes. Ethics board approval and patient consent were not required due to the nature of a systematic review. This meta-analysis was performed according the guidelines of Preferred Reporting Items for Systematic reviews and Meta-Analyses (PRISMA).

### Definitions and outcome

The primary outcome was 28-day mortality and secondary outcomes included recovery of renal function, RRT dependence among survivors and adverse events.

### Data analysis

We used the Cochrane Risk of Bias tool to evaluate the quality of enrolled studies and the risk of bias^26^. The statistical analysis was conducted using the software Review Manager, version 5.3. The degree of heterogeneity was evaluated with the *Q* statistic generated from the χ^2^ test. The proportion of statistical heterogeneity was assessed by the *I*^2^ measure. Heterogeneity was considered significant when the *p*-value was less than 0.10 or the *I*^2^ more than 50%. The fixed effects model and the random effects model were applied when the data was homogenous and heterogeneous, respectively. Pooled risk ratios (RR) and 95% confidence intervals (CI) were calculated for outcome analyses. Funnel plot was used to probe for publication bias. A p-value <0.05 was set as the threshold of statistical significance. Sensitivity analyses were conducted by excluding or subgrouping studies to reduce the potential confounding effects of patient population, RRT modality, study design, and study sample size.

## Results

### Study selection and characteristics

**Figure 1:**
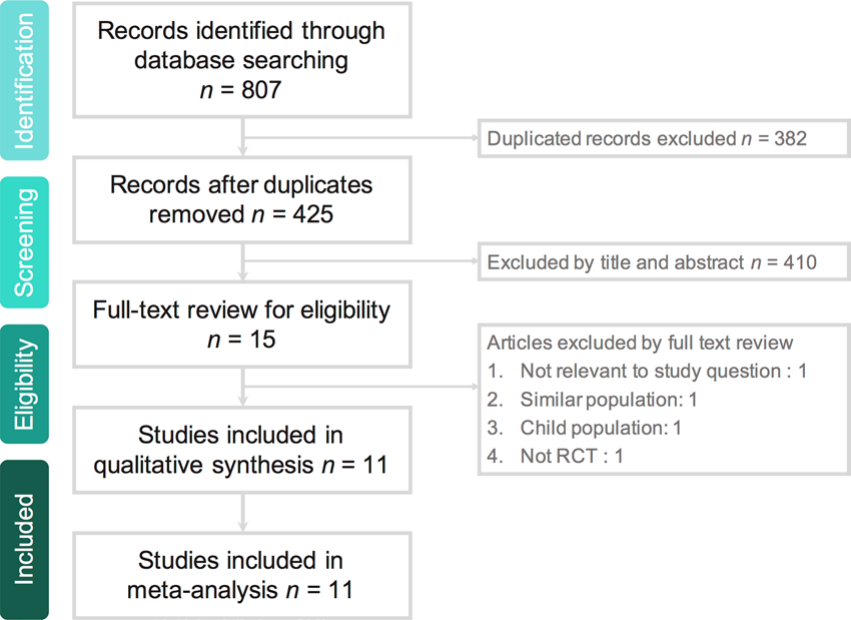
Flowchart of the study selection for the meta-analysis.

**Figure 2:**
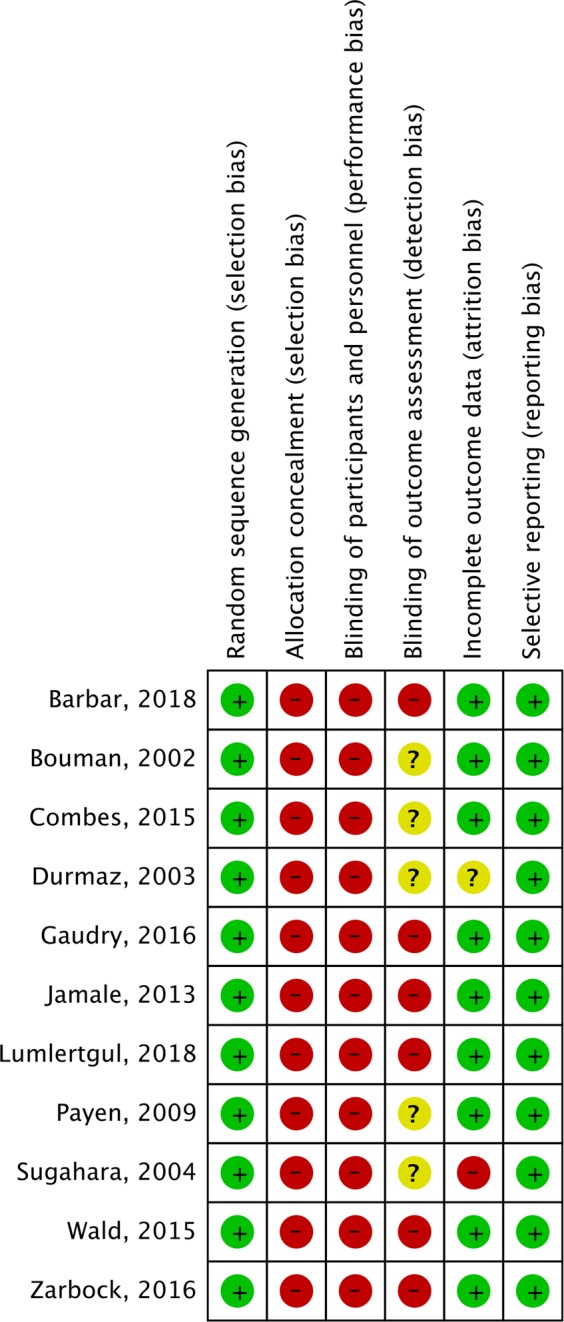
Risk of bias in each study and domain.

The search program yielded 807 references, including 207 from Pubmed, 325 from Embase, and 274 from the Cochrane database. Then, 425 articles were screened for title and abstract after excluding 382 duplicated articles. Finally, a total of eleven RCTs^15–24,27^ fulfilling the inclusion criteria were included in this meta-analysis (Fig. 1, Supplemental Table 2). All the studies^15–24^ were designed to compare the clinical efficacy of early and late RRT for critically ill patients with AKI (Table 1). During the initial enrollment, early and late RRT was applied for 1131 and 1111 patients, respectively. Four studies^17,18,20,23^ were conducted in a single center, and other seven were multicenter studies^15,16,19,21,22,24,27^. Six studies were performed in Europe^15,16,19,22–24^, four studies were conducted in Asia^17,18,20,27^, and one study^21^ was done in North America. The modalities of RRT varied, including mixed intermittent hemodialysis (IHD)/CRRT in three studies^21,22,24^, CRRT only in six studies^15,16,19,20,23,27^, and IHD only in two studies^17,18^. Two studies^19,24^ only enrolled patients with sepsis, and more than half of enrolled patients had sepsis in another three studies^21,22,27^. Figure 2 show the analyses of risk of bias. The risk of allocation concealment, the risk of blinding of participants and personnel, and the risk of blinding of outcome assessment were classified as high or unclear.

**Table 1:** Characteristics of enrolled studies.

Author, year	Setting	Study period	Nation	Site	Mode	Number (%) of patients with sepsis	Mean age		No of patients		Male sex (%)		SOFA scores	
							Early	Late	Early	Late	Early	Late	Early	Late
Bouman, 2002	Mixed	1998–2000	Netherland	M	CRRT	NA	69	67	70	36	59.0	61	10.2	10.6
Durmaz, 2003	Surgical	1999–2001	Turkey	S	IHD	2 (4.5)	58	54	21	23	76	83	NA	NA
Sugahara, 2004	Surgical	1995–1997	Japan	S	CRRT	NA	65	64	14	14	64	64	NA	NA
Payen, 2009	Mixed	1997–2000	France	M	CRRT	76 (100)	58	59	37	39	73	69	11.6	10.4
Jamale, 2013	Mixed	2010–2012	India	S	IHD	44 (21)	43	42	102	106	61	75	7.6	8.2
Combes, 2015	Surgical	2009–2012	France	M	CRRT	NA	61	58	112	112	79	80	11.5	12
Wald, 2015	Mixed	2012–2013	Canada	M	IHD/CRRT/SLED	56 (56)	62	64	48	52	73	71	12	11.9
Zarbock, 2016	Surgical	2013–2015	Germany	S	CRRT	75 (32)	66	68	112	119	70	57	15.6	16
Gaudry, 2016	Mixed	2013–2016	France	M	IHD/CRRT	483 (78)	65	67	311	308	67	64	10.9	10.8
Lumlertgul, 2018	Mixed	2016–2017	Thailand	M	CRRT	69 (58.5)	68	67	58	60	50	48	12.7	11.4
Barbar, 2018	Mixed	2012–2016	France	M	IHD/CRRT	488 (100)	70	69	246	242	58	64	12.3	13
Author, year	Definition of early renal replacement therapy													
Bouman, 2002	Dialysis initiated within 12 h after fulfilling the following criteria: urine output < 30 mL/h and Cr clearance < 20 mL/min on 3-h sample													
Durmaz, 2003	Prophylactic perioperative hemodialysis in patients with nondialysis-dependent moderate (serum creatinine > 2.5 mg/dL) renal dysfunction undergoing coronary artery bypass surgery													
Sugahara, 2004	3 h urine output < 30 ml/hr													
Payen, 2009	96 hours of isovolemic CVVH in addition to standard sepsis management, start within 24 hours after randomization,													
Jamale, 2013	Serum urea nitrogen and/or creatinine levels increased to 70 and 7 mg/dL, respectively													
Combes, 2015	Urine output > 1500 mL/h and Epinephrine (E) < 0.1 ug/kg/min and Norepinephrine (NE) < 0.2 ug/kg/min and E + (NE/2) < 0.1 ug/kg/min													
Wald, 2015	Patients started RRT within 12 h of fulfilling eligibility													
Zarbock, 2016	KDIGO Stage 2 AKI (within 8 h) and plasma neutrophil gelatinase–associated lipocalin level higher than 150 ng/mL													
Gaudry, 2016	KDIGO Stage 3 AKI (within 6 h)													
Lumlertgul, 2018	FST-nonresponsive patients (urine output less than 200 mL in 2 h) (initiation within 6 h)													
Barbar, 2018	Within 12 hours after documentation of failure-stage acute kidney injury of RIFLE classification system													

AKI, acute kidney injury; CRRT, continuous renal replacement therapy; FST, furosemide stress test; IHD, intermittent hemodialysis; KDIGO, Kidney Disease: Improving Global Outcomes; RIFLE, risk, injury, failure, loss, and end-stage kidney disease; SLED, sustained low efficiency dialysis; NA, not available; S, single center; M, multicenter.

### Primary outcomes

**Figure 3:**
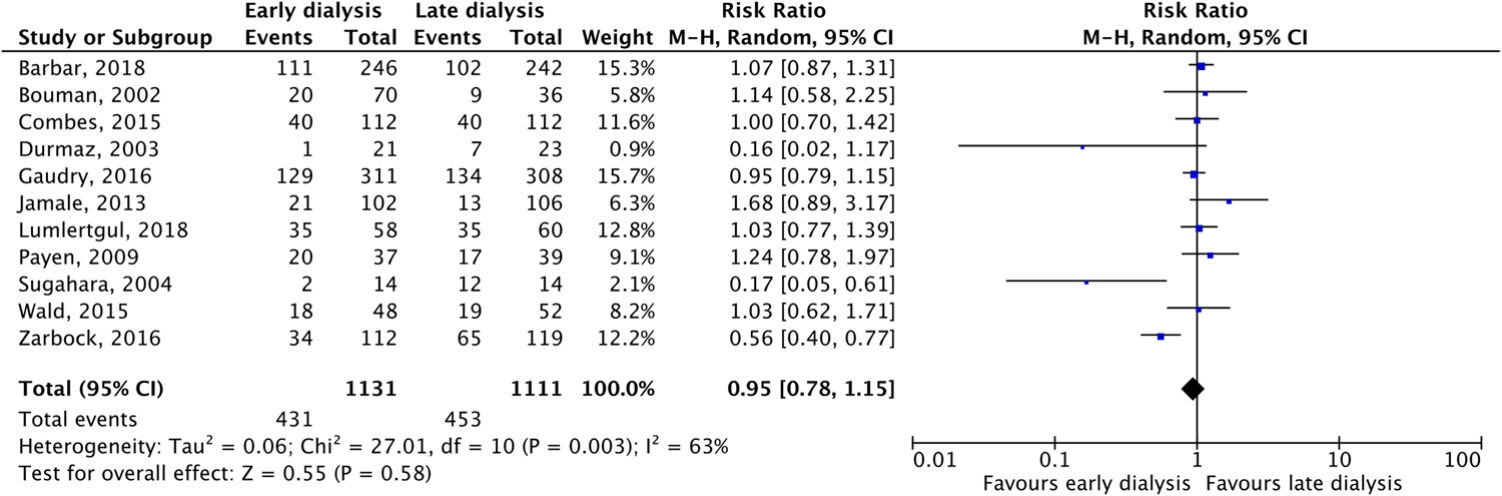
Forest plot for 28-day mortality.

**Figure 4:**
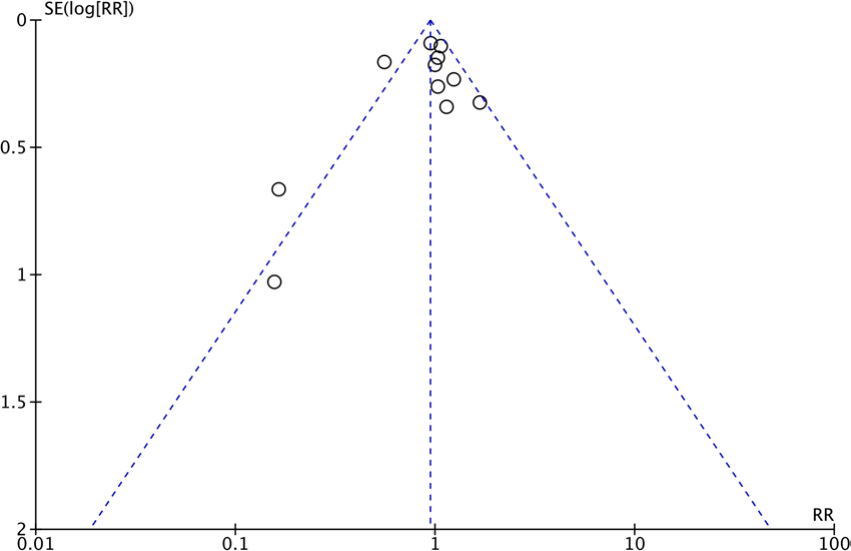
Funnel plot for 28-day mortality.

In the eleven enrolled trials, the pooled 28-day mortality was 38.1% (431/1131) and 40.7% (453/1111) in the groups of patients assigned to early and late RRT, respectively, with no significant difference between groups (RR, 0.95; 95% CI, 0.78–1.15, *I*^2^ = 63%, Fig. 3). Sensitivity analysis after deleting an individual study each time to reflect the influence of the single dataset on the pooled RR showed similar findings. The publication bias was shown in funnel plot (Fig. 4). We found no differences between early and late RRT in terms of 60-day mortality RR, 0.96; 95% CI, 0.75–1.23, *I*^2^ = 31%) in four studies^16,22–24^, and 90-day mortality (RR, 0.97 95% CI, 0.64–1.45, *I*^2^ = 0%) in four studies^16,21,23,24^. Four studies reported the ICU mortality^15,16,21,24^ and five studies^15–18,21^ showed in-hospital mortality. The pooled ICU mortality (RR, 1.16; 95% CI, 0.88–1.52, *I*^2^ = 0%) and in hospital-mortality (RR, 1.25; 95% CI, 0.74–2.11, *I*^2^ = 49%) were similar between groups.

In the four studies^16,17,20,23^ that only enrolled surgical patients, the early RRT group had a lower risk of mortality than the late RRT group (RR, 0.52; 95% CI, 0.27–0.99, *I*^2^ = 77%). There were no significant differences in terms of mortality between groups in the analysis of other subgroups, including mixed study populations, study sites, study designs, portion of patients with sepsis and RRT modality (Table 2).

**Table 2:** Subgroup analysis.

Subgroup	No of study	No of patients		Random-effect model		*I *^2^ (%)	Test of heterogeneity P
		Early RRT	Late RRT	Risk Ratio	95% CI		
**Study population**							
Surgical	4	259	268	0.52	0.27–0.99	77	0.005
Mixed	7	872	843	10.4	0.93–1.17	0	0.71
**Study design**							
Single center	4	249	262	0.51	0.20–129	81	0.001
Multicenter	7	882	849	1.02	0.91–1.14	0	0.96
**Modality of RRT**							
IHD only	2	123	129	0.62	0.06–6.53	80	0.02
CRRT only	6	403	380	0.85	0.59–1.21	73	0.002
Mixed	3	605	602	1.01	0.88–1.15	0	0.70
**Portion of patients with sepsis**							
100%	2	283	281	1.10	0.91–1.32	0	0.57
>50–<100%	3	417	420	0.98	0.84–1.14	0	0.88
**Study site**							
Europe	6	888	856	0.94	0.76–116	63	0.02
Asia	4	195	203	0.66	0.28–1.56	78	0.003
North America	1	48	52	1.03	0.62–1.71	NA	NA

### Secondary outcomes

**Figure 5:**
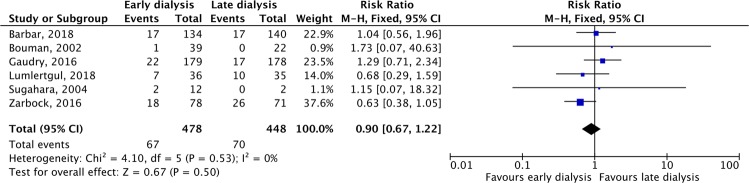
Forest plot for dialysis-dependence among survivors on day 28.

**Figure 6:**
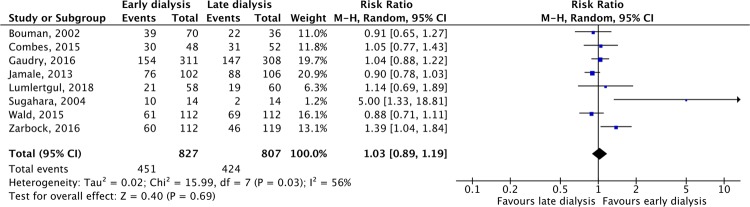
Forest plot for renal function recovery.

Six studies^15,20,22–24,27^ reported the rate of RRT dependence in survivors on day 28, and no significant difference was found between early and late RRT groups (RR, 0.90; 95% CI, 0.67–1.25, *I*^2^ = 0%, Fig. 5). The five studies^16,18,21,23,24^ that reported the rate of RRT dependence on day 90, showed similar rates in the two groups (RR, 0.76; 95% CI, 0.30–1.90, *I*^2^ = 0%). The recovery of renal function was reported in eight studies^15,16,18,20–23,27^, with similar rates in the two groups (RR, 1.03; 95% CI, 0.89–1.19, *I*^2^ = 56%, Fig. 6).

### Adverse events

We assessed the rates of several common adverse events during RRT including hemorrhage, hypotension, arrhythmia, catheter-related infection, hypokalemia, hyperkalemia, metabolic acidosis and hypophosphatemia. A pooled analysis of five studies^15,18,21,22,27^ reporting the risk of catheter-related infections showed the early RRT group had a higher risk of this infection than late RRT group (RR, 1.7, 95% CI, 1.01–2.97, *I*^2^ = 0%) The early RRT group had a higher risk of hypophosphatemia than the late RRT group in a pooled analysis of 3 studies^16,22,27^ (RR, 2.5, 95% CI, 1.25–4.99, *I*^2^ = 77%). There were no significant differences between early and late groups in terms of hemorrhage (RR, 0.88, 95% CI, 0.68–1.14, *I*^2^ = 0%) in seven studies^15,16,18,21–24^, hypotension (RR, 1.11, 95% CI, 0.96–1.29, *I*^2^ = 0%) in six studies^16,18,21,23,24,27^, arrhythmia (RR, 1.21, 95% CI, 0.83–1.77, *I*^2^ = 36%) in six studies^17,21–24,27^, hypokalemia (RR, 1.11, 95% CI, 0.83–1.47, *I*^2^ = 24%) in three studies^16,22,27^, and hyperkalemia (RR, 0.52, 95% CI, 0.17–1.61, *I*^2^ = 57%) in three studies^16,22,24^. Pooled analysis of two studies^16,24^ showed that the early RRT group had a lower risk of metabolic acidosis than the late RRT group (RR, 0.60, 95% CI, 0.39–0.90, *I*^2^ = 0%).

## Discussion

This meta-analysis of eleven RCTs with 1131 and 1111 AKI patients receiving early and late RRT, respectively, provided several significant findings. Most importantly, early RRT was not associated with a better outcome for these patients than late RRT. Overall, there was no significant difference in 28-day mortality between groups. There were no differences with different study sites (Europe, Asia or North America), study designs (single or multi-center), portion of patients with sepsis (50–<100%, or 100%) or RRT modality (IHD, CRRT, mixed). The early and late RRT groups had similar ICU-, hospital-, 60 day- and 90 day- mortality rates. All these findings are consistent with previous meta-analyses^25,28–30^, and indicate that early RRT does not provide additional survival benefits for AKI patients compared with late RRT. In addition to mortality, Pasin *et al*.^25^ ever showed that early RRT was associated with a significant reduction in length of hospital stay. However, the positive impact of early RRT on the length of hospital stay still need further confirmation in the high-quality studies.

Subgroup analysis of four studies^16,17,20,23^ that only enrolled surgical patients showed that the early RRT group had a lower risk of mortality than the late RRT group (RR, 0.52; 95% CI, 0.27–0.99, *I*^2^ = 77%). This finding is consistent with the result of a previous meta-anlaysis^31^ of nine retrospective cohort studies and two RCTs showing a lower 28-day mortality rate the early RRT group (OR = 0.29, 95% CI, 0.16–0.52, p < 0.0001) than the late RRT group among critically ill patients with AKI after cardiac surgery. However, both that meta-analysis^31^ and our findings in surgical patients were based on studies with very high heterogeneity. Further research with a larger number of studies and consistent results is still needed to confirm this finding in surgical patients.

We also found no differences in the recovery of renal function or RRT dependency in the early and late RRT groups. In Karvellas *et al*.’s meta-analysis^32^ of 15 studies, early RRT was associated with greater renal recovery than late RRT. However, only two RCTs were enrolled in that meta-analysis^32^, and the quality of those heterogeneous studies varied. In contrast, the present analysis only enrolled large-scale RCTs, and our findings were consistent with other meta-analyses^29,30,33^ of RCTs. These results should be more convincing than Karvellas *et al*.'s meta-analysis^32^. Therefore, based on current evidence, early RRT was not associated with greater renal recovery and lower dialysis dependence than late RRT for critically ill patients with AKI.

We cannot omit another important issue of RRT – safety. We evaluated the risks of several common complications during RRT. Although the incidence of most adverse events such as hemorrhage, hypotension, arrhythmia, hypokalemia and hyperkalemia were similar between groups, the early RRT group had higher risks of catheter-related infections, and hypophosphatemia than the late RRT group. Overall, our findings should remind clinicians to keep alert concerning the high risks of these two complications in early RRT for patients with AKI.

Although this meta-analysis enrolled several large-scale RCTs with a reasonable quality to enhance the level of evidence, there was one major limitation. There was relatively high heterogeneity with an *I*^2^ value of more than 50% in the outcome analysis. These heterogeneities could be caused by significant variations in the study design, population characteristics, disease severity, timing of initiating RRT, modality of RRT, and duration of follow-up in the studies.

## Conclusion

This meta-analysis suggested that early RRT does not improve the survival, RRT dependence, or renal function recovery of critically ill patients with AKI in comparison with late RRT. Early RRT was associated with a lower 28-day mortality than late RRT in surgical patients with AKI. However, clinicians should be vigilant as early RRT can carry higher risks of catheter-related infection and hypophosphatemia during dialysis than late RRT.

## Supplementary information

Supplemental tables

## Data Availability

The datasets used and/or analyzed in the current study are available from the corresponding author upon request.
